# Supplementary material for: The origin of a primordial genome through spontaneous symmetry breaking
Source: Nat Commun. 2017 Aug 15;8:250. doi: 10.1038/s41467-017-00243-x (PMC5557888; doi:10.1038/s41467-017-00243-x)
Supplement: Supplementary file 1 — Supplementary Information [file 41467_2017_243_MOESM1_ESM.pdf]

File name: Supplementary Information
Description: Supplementary figures, supplementary methods and supplementary references.

File name: Peer review file
Description:

# Supplementary Methods

## The minimal model displaying symmetry breaking

We hypothesised the three conditions sufficient for symmetry breaking as described in the main text (under 'Sufficient conditions for functional symmetry breaking'). To test that hypothesis, we drastically simplified the model chiefly discussed in the main text (the main model, for short) and constructed a model that minimally satisfies those conditions (the minimal model, for short). The details of the minimal model are described in this section (for the details of the main model, see Methods under 'The implementation of the model' in the main text).

The minimal model is formulated as a hierarchical Moran process. It consists of $N$ replicators, which are partitioned into groups. In each time step, one replicator is chosen for replication with a probability proportional to its fitness defined below, and one replicator is chosen for removal with a probability $1/N$. A replicator can be chosen simultaneously for both replication and removal, in which case replication precedes removal. If the number of replicators in a protocell exceeds $V$, the protocell undergoes binary fission with its internal replicators randomly distributed between the daughter cells. Note that this minimal model abstracts away the features of the main model that are specific to RNA replicators and are considered unnecessary for symmetry breaking (namely, it ignores substrates, complementarity, and complex formation).

The fitness of replicators is determined by the degree of cooperation among replicators. The cooperativeness of a replicator is determined by two numerical parameters assigned to each replicator (denoted by $k_x$, where $x \in \{1, 2\}$). The values of $k_x$ determine the fitness of a replicator (denoted by $f$) as follows:

$$f(k_1, k_2; \bar{k}_1, \bar{k}_2) = e^{\bar{k}_1 + \bar{k}_2 - r(k_1 + k_2)}, \tag{1}$$

where a bar denotes an average taken over all replicators in the same protocell, and $r$ is a constant indicating the cost of cooperation ($0 < r < 1$).

Molecular-level evolution tends to minimise $k_1$ and $k_2$ because the selection gradient at the molecular level is negative:

$$\partial_{k_x} \ln f = -r < 0, \tag{2}$$

where $\partial_{k_x}$ denotes a partial derivative with respect to $k_x$, and $\bar{k}_x$ is considered independent of $k_x$.

Conversely, cellular-level evolution tends to maximise $\bar{k}_1$ and $\bar{k}_2$ because the selection gradient at the cellular level is positive, as shown below. Let the fitness of a protocell be defined as the average fitness of replicators in the protocell, i.e., $\overline{f(k_1, k_2; \bar{k}_1, \bar{k}_2)}$. The fitness of a protocell can be approximated by

$$\overline{f(k_1, k_2; \bar{k}_1, \bar{k}_2)} \approx f(\bar{k}_1, \bar{k}_2; \bar{k}_1, \bar{k}_2) \tag{3}$$

under the assumption that the variance of $k_x$ within a protocell (denoted by $\sigma^2_{k_x}$) is sufficiently small and that the covariance between $k_1$ and $k_2$ within a

protocell (denoted by $\sigma_{k_1,k_2}$) is zero (the latter assumption is reasonable if $k_1$ and $k_2$ are mutationally independent). Using the above approximation, the selection gradient at the cellular level is calculated as

$$\partial_{\bar{k}_x} \ln \bar{f} \approx 1 - r > 0. \tag{4}$$

Because the gradient is positive, protocells tend to evolve toward maximising $\bar{k}_x$.

When a new replicator is produced through replication, its $k_x$ values are copied from the replicator serving as its template with possible mutation (complementarity was ignored). The values of $k_x$ were mutated by three different methods, which have distinct effects on the way the rate of molecular-level evolution depends on the value of $\bar{k}_x$. To describe these effects in the following sections, we here note that the rate of molecular-level evolution—i.e., the change of $\bar{f}$ per generation (denoted by $\Delta\bar{f}$)—is approximated by

$$\Delta\bar{f} \approx \sum_{x=1}^{2} \bar{f}\sigma_{k_x}^2 (1 + \partial_{k_x} \ln f)\partial_{k_x} \ln f, \tag{5}$$

where the derivatives are evaluated at $k_x = \bar{k}_x$ (see the section under 'The derivation of Equation (5)' below). Note that

$$\Delta\bar{f} \leq 0 \tag{6}$$

because $\bar{f} > 0$, $\sigma_{k_x}^2 \geq 0$, and $-1 < \partial_{k_x} \ln f < 0$.

### The first method of mutating $k_x$

This method is identical to the default method of mutation in the main model. Namely, $k_x$ is mutated by adding a number randomly drawn from a uniform distribution on the interval $(-\delta, \delta)$. The value of $k_x$ is bounded above with a reflecting boundary ($k_x \leq 5$ unless otherwise stated); however, it is not bounded below (this boundary condition reflects the fact that there are far more genotypes with low fitness than those with high fitness). According to this boundary condition, the value of $k_x$ can be negative, in which case, however, $k_x$ is regarded as zero in calculating $f$. Consequently,

$$\partial_{k_x} \ln f = \begin{cases} -r & \text{if } k_x > 0, \\ 0 & \text{if } k_x < 0. \end{cases} \tag{7}$$

According to Equations (5) and (7), the rate of molecular-level evolution $|\Delta\bar{f}|$ decreases if either $\bar{k}_1$ or $\bar{k}_2$ decreases below zero. Therefore, symmetry breaking is expected (see Supplementary Fig. 7).

### The second method of mutating $k_x$

This method is the same as described above, except that the special rule that applies when $k_x < 0$ is removed (note that this is impossible in the main model

because $k_{xy}$ in that model denotes a rate constant). According to this method,

$$\partial_{k_x} \ln f = -r \tag{8}$$

irrespective of the value of $k_x$. According to Equations (5) and (8), $\Delta \bar{f}$ is invariant with respect to $\bar{k}_x$. Therefore, symmetry breaking is not expected (see Supplementary Fig. 9).

### The third method of mutating $k_x$

In this method, each replicator is assigned numerical parameters representing a genotype (denoted by $g_x$, where $x \in \{1, 2\}$). The value of $k_x$ (phenotype) is defined as a logistic function of $g_x$ as follows:

$$k_x = \frac{1}{1 + \exp(-g_x)}. \tag{9}$$

The value of $g_x$ is mutated by adding a number randomly drawn from a uniform distribution on the interval $(-\delta, \delta)$. The value of $g_x$ is bounded above with a reflecting boundary, but not bounded below ($g_i \leq 5$ unless otherwise stated). In this case,

$$\sigma^2_{k_x} \approx (\partial_{g_x} k_x)^2 \sigma^2_{g_x} \to 0 \quad \text{as} \quad \bar{g}_x \to -\infty \tag{10}$$

because $\partial_{g_x} k_x \to 0$. According to Equations (5) and (10), the rate of molecular-level evolution $|\Delta \bar{f}|$ decreases as $\bar{k}_x$ decreases. Therefore, symmetry breaking is expected (see Supplementary Fig. 8).

## The model incorporating the RNA-folding genotype-phenotype map

This section describes the model incorporating a complex genotype-phenotype map of replicators based on the RNA folding algorithm outlined in the section under 'RNA folding genotype-phenotype map' in the main text.

The model is the same as that described in the previous section, except for the definition of $k_x$ as described below. Each replicator is assigned a pair of complementary RNA sequences of 50 bases (denoted by $s_x$ where $x \in \{\mathrm{P}, \mathrm{M}\}$). The secondary structure of $s_x$ (denoted by $p_x$) is computed with Vienna RNA Package 2.0 [1]. $k_x$ is defined as a function of $s_x$, via $p_x$, as follows:

$$k_x = \begin{cases} 1 - D(p_x, p_0)/10 & \text{if } D(p_x, p_0) < 10, \\ 0 & \text{else,} \end{cases} \tag{11}$$

where $p_0$ is a target structure (which is chosen arbitrary and fixed throughout a simulation), and $D(p_x, p_0)$ is the distance between $p_x$ and $p_0$ defined as the minimum number of base pairs that must be paired and unpaired to transform $p_x$ into $p_0$.

When a new replicator is produced through replication, its sequences are copied from the template with possible point mutations. The probability of mutation is $m/100$ per base per replication (thus, the probability of mutation per genome per replication is approximately $m$).

The results obtained with this model are shown in Supplementary Figs. 10 and 11.

## The derivation of Equation (5)

Let the frequency of a replicator with $k_1$ and $k_2$ be $P(k_1, k_2)$. The fitness of a protocell $\bar{f}$ is

$$\bar{f} = \int f(k_1, k_2; \bar{k}_1, \bar{k}_2) P(k_1, k_2) dk_1 dk_2. \tag{12}$$

The frequency of a replicator with $k_1$ and $k_2$ in the next generation, denoted by $P'(k_1, k_2)$, is

$$\begin{aligned} P'(k_1, k_2) &= \frac{f(k_1, k_2; \bar{k}_1, \bar{k}_2) P(k_1, k_2)}{\int f(k_1, k_2; \bar{k}_1, \bar{k}_2) P(k_1, k_2) dk_1 dk_2} \\ &= \bar{f}^{-1} f(k_1, k_2; \bar{k}_1, \bar{k}_2) P(k_1, k_2). \end{aligned} \tag{13}$$

Thus, the fitness of a protocell in the next generation is

$$\begin{aligned} \bar{f}' &= \int f(k_1, k_2; \bar{k}'_1, \bar{k}'_2) P'(k_1, k_2) dk_1 dk_2 \\ &= \bar{f}^{-1} \int f(k_1, k_2; \bar{k}'_1, \bar{k}'_2) f(k_1, k_2; \bar{k}_1, \bar{k}_2) P(k_1, k_2) dk_1 dk_2, \end{aligned} \tag{14}$$

where $\bar{k}'_x$ is the average value of $k_x$ in the next generation. Using the notation

$$\Delta_{\bar{k}_x} f = f(k_1, k_2; \bar{k}'_1, \bar{k}'_2) - f(k_1, k_2; \bar{k}_1, \bar{k}_2), \tag{15}$$

we can write $\bar{f}'$ as

$$\begin{aligned} \bar{f}' = \bar{f}^{-1} \Bigg( &\int f^2(k_1, k_2; \bar{k}_1, \bar{k}_2) P(k_1, k_2) dk_1 dk_2 \\ &+ \int f(k_1, k_2; \bar{k}_1, \bar{k}_2) \Delta_{\bar{k}_x} f P(k_1, k_2) dk_1 dk_2 \Bigg) \\ = \bar{f}^{-1} &\left( \overline{f^2} + \overline{f \Delta_{\bar{k}_x} f} \right) \end{aligned} \tag{16}$$

Therefore, the change in the fitness of a protocell $\bar{f}' - \bar{f}$ (denoted by $\Delta \bar{f}$) is

$$\Delta \bar{f} = \bar{f}^{-1} \left( \sigma_f^2 + \overline{f \Delta_{\bar{k}_x} f} \right), \tag{17}$$

where $\sigma_f^2$ is the variance of the fitness of replicators within a protocell. This is the Price equation [2].

The variance $\sigma_f^2$ can be approximated by

$$\begin{aligned}\sigma_f^2 &\approx \overline{[f(k_1,k_2;\bar{k}_1,\bar{k}_2) - f(\bar{k}_1,\bar{k}_2;\bar{k}_1,\bar{k}_2)]^2} \\ &\approx \overline{[(k_1-\bar{k}_1)\partial_{k_1}f + (k_2-\bar{k}_2)\partial_{k_2}f]^2} \\ &= \sigma_{k_1}^2(\partial_{k_1}f)^2 + \sigma_{k_1}^2(\partial_{k_2}f)^2,\end{aligned} \tag{18}$$

where the derivatives are evaluated at $k_x = \bar{k}_x$ (assuming that $\sigma_{k_x}^2 \ll 1$ and $\sigma_{k_1,k_2} = 0$ as before).

To calculate $\overline{f\Delta_{\bar{k}_x}f}$, we need to calculate the change of $\bar{k}_x$ through the evolution of replicators in a protocell (denoted by $\Delta\bar{k}_x$). According to the Price equation [2],

$$\Delta\bar{k}_x = \bar{f}^{-1}\left(\sigma_{f,k_x} + \overline{f\Delta_\delta k_x}\right), \tag{19}$$

where $\sigma_{f,k_x}$ is the covariance between $f$ and $k_x$, and $\Delta_\delta k_x$ is the mean change of $k_x$ due to mutation. The covariance $\sigma_{f,k_x}$ can be approximated under the assumptions that $\sigma_{k_x}^2$ is sufficiently small and that $\sigma_{k_1,k_2} = 0$:

$$\sigma_{f,k_x} \approx \sigma_{k_x}^2 \partial_{k_x} f. \tag{20}$$

Moreover,

$$\overline{f\Delta_\delta k_x} = 0 \tag{21}$$

under the assumption that mutation is unbiased. Combining these equations, we obtain

$$\Delta\bar{k}_x \approx \sigma_{k_x}^2 \partial_{k_x} \ln f. \tag{22}$$

Using Equation (22), $\Delta_{\bar{k}_x}f$ can be approximated by

$$\begin{aligned}\Delta_{\bar{k}_x}f &\approx (\partial_{\bar{k}_1}f)\Delta\bar{k}_1 + (\partial_{\bar{k}_2}f)\Delta\bar{k}_2 \\ &= f\Delta\bar{k}_1 + f\Delta\bar{k}_2 \quad (\text{using } \partial_{\bar{k}_x}f = f) \\ &= f\sigma_{k_1}^2\partial_{k_1}\ln f + f\sigma_{k_2}^2\partial_{k_2}\ln f\end{aligned} \tag{23}$$

under the assumption that $\Delta\bar{k}_x$ is sufficiently small. Using the above equation, we obtain

$$\begin{aligned}\overline{f\Delta_{\bar{k}_x}f} &\approx \overline{f^2}(\sigma_{k_1}^2\partial_{k_1}\ln f + \sigma_{k_2}^2\partial_{k_2}\ln f) \\ &= (\sigma_f^2 + \bar{f}^2)(\sigma_{k_1}^2\partial_{k_1}\ln f + \sigma_{k_2}^2\partial_{k_2}\ln f).\end{aligned} \tag{24}$$

Substituting Equations (24) and (18) into Equation (17), we obtain

$$\begin{aligned}\Delta\bar{f} = \bar{f}^{-1}\,&\big[\sigma_{k_1}^2(\partial_{k_1}f)^2 + \sigma_{k_2}^2(\partial_{k_2}f)^2 \\ &+ \{\sigma_{k_1}^2(\partial_{k_1}f)^2 + \sigma_{k_2}^2(\partial_{k_2}f)^2 + \bar{f}^2\}\{\sigma_{k_1}^2\partial_{k_1}\ln f + \sigma_{k_2}^2\partial_{k_2}\ln f\}\,\big].\end{aligned} \tag{25}$$

The terms involving $(\sigma_{k_x}^2)^2$ can be ignored because $\sigma_{k_x}^2 \ll 1$ has been assumed:

$$\Delta\bar{f} \approx \sum_{x=1}^{2} \bar{f}^{-1}[\sigma_{k_x}^2(\partial_{k_x}f)^2 + \bar{f}^2\sigma_{k_x}^2\partial_{k_x}\ln f] \tag{26}$$

The above equation can be transformed into Equation (5) by substituting $\partial_{k_1}f = f\partial_{k_1}\ln f$.

## Supplementary References


[1] Ronny Lorenz, Stephan H. Bernhart, Christian Höner zu Siederdissen, Hakim Tafer, Christoph Flamm, Peter F. Stadler, and Ivo L. Hofacker. ViennaRNA Package 2.0. *Algorithms for Molecular Biology*, 6:26, 2011. doi: 10.1186/1748-7188-6-26.

[2] George R. Price. Selection and covariance. *Nature*, 227:520–521, 1970. doi: 10.1038/227520a0.

# Supplementary Figures

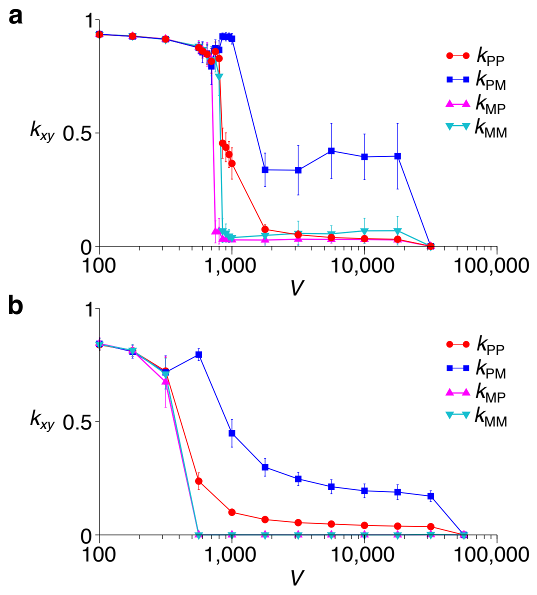


Supplementary Figure 1: Symmetry breaking in the alternative models of mutation. The equilibrium average catalytic activities ($k_{xy}$) are plotted as functions of cell size ($V$). The values of $k_{xy}$ were first averaged over all replicators at each time point. Then, the average (symbols) and s.d. (error bars) of $k_{xy}$ over time were calculated after equilibration. **a.** The model in which $k_{xy}$ is bounded below by zero with a reflecting boundary. **b.** The model in which $k_{xy}$ is mutated in a logarithmic scale. The parameters were the same as in Fig. 2.

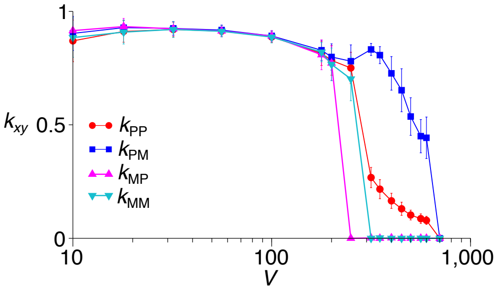


Supplementary Figure 2: Symmetry breaking in the model incorporating the continual emergence through mutation of parasitic replicators providing no catalysis. The equilibrium average catalytic activities ($k_{xy}$) are plotted as functions of cell size ($V$). The values of $k_{xy}$ were first averaged over all replicators at each time point. Then, the average (symbols) and s.d. (error bars) of $k_{xy}$ over time were calculated after equilibration. In this model, mutation occurs with probability $m$ per replication. With probability 0.1, mutation sets all the $k_{xy}$ values of a replicator to zero; with probability 0.9, it changes the $k_{xy}$ values of a replicator by the default method described in the main text. The parameters were the same as in Fig. 2.

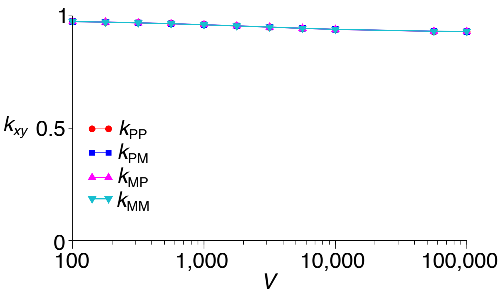


Supplementary Figure 3: The absence of symmetry breaking in the model assuming one-step replication (instead of two-step replication). The equilibrium average $k_{xy}$ values are plotted as functions of cell size ($V$). The values of $k_{xy}$ were first averaged over all replicators at each time point. Then, the average (symbols) and s.d. (error bars) of $k_{xy}$ over time were calculated after equilibration. In this model, replication occurs without explicitly involving complex formation. Accordingly, the $k_{xy}$ values were re-defined as the rates of replication. Replication was modeled as a third-order chemical reaction involving three particles: a replicator serving as a catalyst, a replicator serving as a template, and a substrate. Specifically, the reaction algorithm was modified as follows. After the first and second particles (denoted by $X$ and $Y$) are chosen as in the original algorithm, a third particle ($Z$) is randomly chosen from the protocell containing $X$ and $Y$ (see Methods under 'The implementation of the model' in the main text for the notation). Replication can occur if and only if two particles are replicators and one is a substrate. For example, if $X$ and $Z$ are replicators and $Y$ is a substrate, $Z$ replicates $X$ with a probability $\alpha\beta k_{zx}^{Z}$, and $X$ replicates $Z$ with a probability $\alpha\beta k_{xz}^{X}$. Moreover, $X$ decays with a probability $\alpha d$ if $X$ is a replicator ($Y$ and $Z$ do not decay). The value of $\alpha$ was chosen such that the inequality $\alpha(2\beta k_{\max} + d) \leq 1$ always holds, where $k_{\max}$ is the maximum possible value of $k_{xy}$ ($k_{\max} = 1$ in this figure). The value of $\beta$ was set to $1/6$ in order to cancel out the fact that there are six possible orders in which three particles are chosen to react. The other parameters were the same as in Fig. 2. The simulation was run only for $4.8 \times 10^6$ (instead of $\geq 10^7$) time steps for $V = 100,000$.

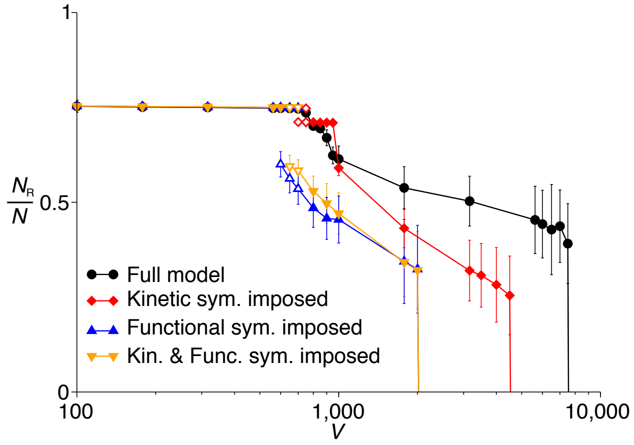


Supplementary Figure 4: The fraction of particles that are replicators ($N_R/N$ where $N_R \equiv N_P + N_M$) as a function of cell size ($V$). The average (symbols) and s.d. (error bars) of $N_R/N$ over time are plotted (calculated after equilibration). The symbols indicate different models: the full model, i.e., the model in which no symmetry is imposed (●); the model in which kinetic symmetry is imposed (⧫); the model in which functional symmetry is imposed (▲); the model in which both kinetic and functional symmetry are imposed (▼). The open symbols indicate metastable states.

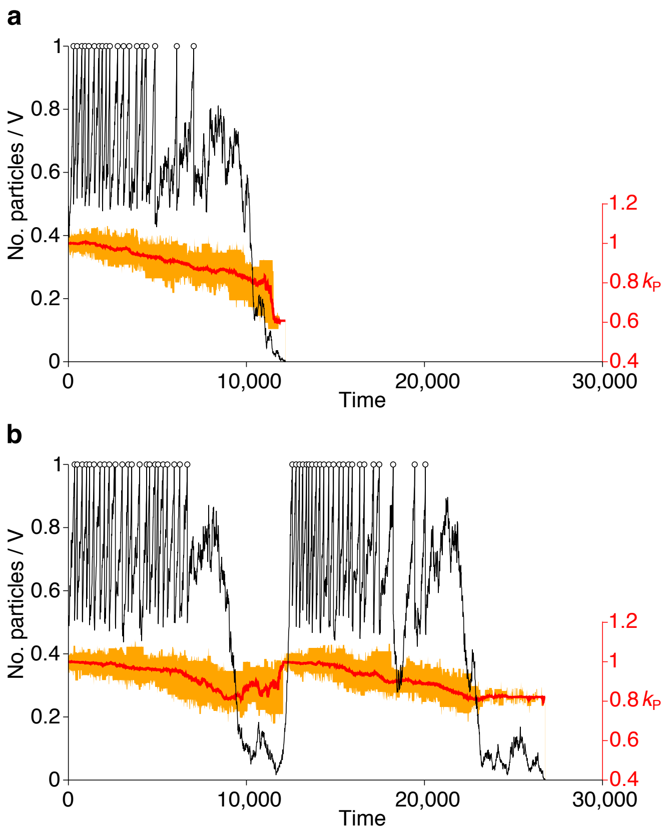


Supplementary Figure 5: The dynamics of a single protocell along its line of descent. Colour coding: the number of particles in a protocell normalised by $V$ ($V = 1700$) (black); cell division (∘); the value of $k_{\mathrm{P}}$ averaged over the replicators in a protocell (red); the range of $k_{\mathrm{P}}$ in a protocell (orange). See Methods for the details of simulations. $k_{\mathrm{M}}$ was set to a negative value so that replicators were functionally asymmetric throughout a simulation. **a.** A protocell ceases reproduction and dies. The average value of $k_{\mathrm{P}}$ steadily decreases owing to molecular-level evolution. **b.** The same simulation as above with a different random seed. A protocell revives its growth (time $\approx$ 12000). The average of $k_{\mathrm{P}}$ abruptly increases before the revival; the range of $k_{\mathrm{P}}$ drastically shrinks, indicating an intracellular population bottleneck.

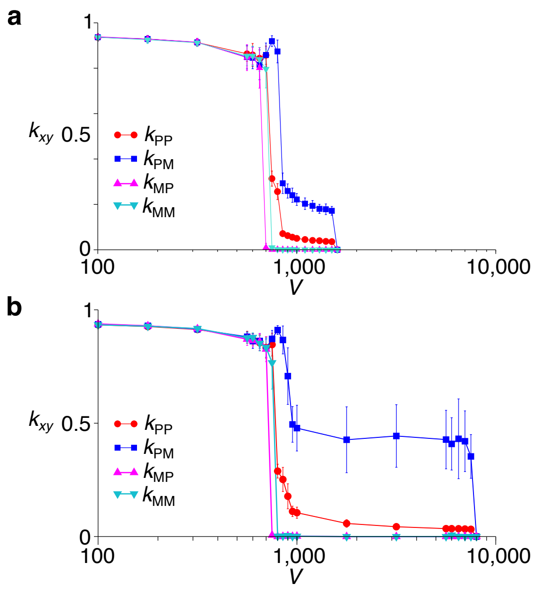


Supplementary Figure 6: The effect of preventing bottleneck-induced growth restoration. The equilibrium average catalytic activities ($k_{xy}$) are plotted as functions of cell size ($V$). The values of $k_{xy}$ were first averaged over all replicators at each time point. Then, the average (symbols) and s.d. (error bars) of $k_{xy}$ over time were calculated after equilibration. **a.** Bottleneck-induced growth restoration was prevented by killing small protocells, i.e., protocells whose cell sizes fell below a threshold $0.1V$. This threshold was set much higher than the minimum number of molecules during population bottlenecks so that growth restoration was prevented (see Supplementary Fig. 5). The killing was implemented by converting all internal molecules of a protocell into substrates so that the total number of molecules and substrates was kept constant. The parameters were the same as in Fig. 2. **b.** For the sake of comparison, the results obtained without preventing bottleneck-induced growth restoration (Fig. 2a) is shown again.

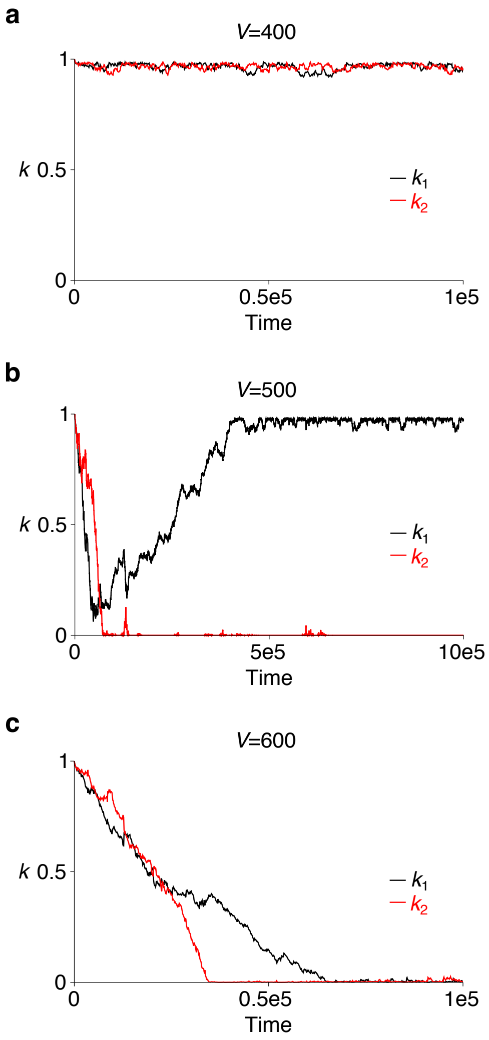


Supplementary Figure 7: Symmetry breaking in the minimal model with the first method of mutation (see Supplementary Methods under 'The first method of mutating $k_x$'). The population averages of $k_x$ values are plotted as functions of time (one unit of time corresponds to $N$ birth-death steps). Colour coding: $k_1$ (black), $k_2$ (red). **a.** $V = 400$. Both $k_1$ and $k_2$ are maximised (i.e., no symmetry breaking). **b.** $V = 500$. The value of $k_1$ is maximised, but that of $k_2$ is minimised (i.e., symmetry breaking). Note that both $k_1$ and $k_2$ first decreases, and $k_1$ starts to increase after $k_2$ decreases to zero. This dynamics is similar to that depicted in Fig. 4. **c.** $V = 600$. Both $k_1$ and $k_2$ are minimised (i.e., no symmetry breaking). Parameters: $m = 0.03$, $\delta = 0.05$, $r = 0.5$, $N = 50V$.

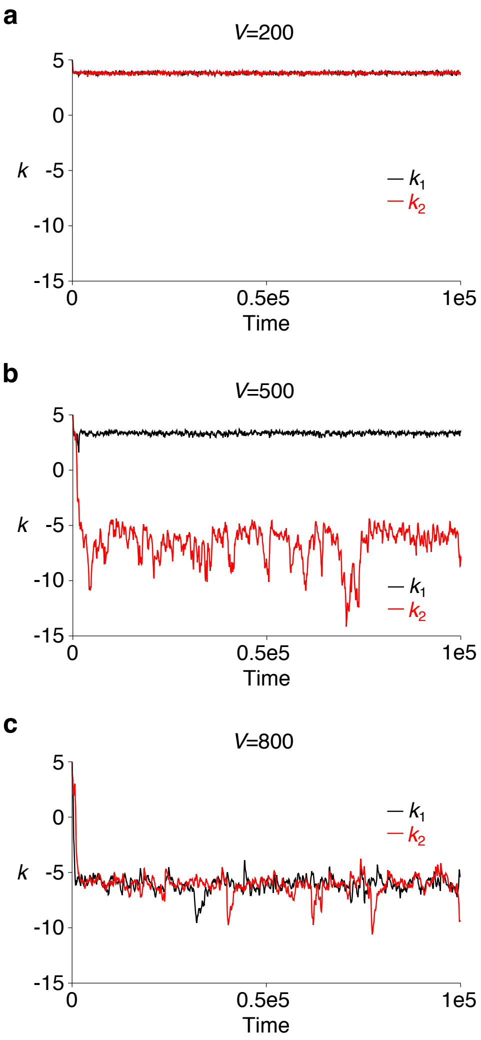


Supplementary Figure 8: Symmetry breaking in the minimal model with the third method of mutation (see Supplementary Methods under 'The third method of mutating $k_x$'). The population averages of $k_x$ values are plotted as functions of time (one unit of time corresponds to $N$ birth-death steps). **a.** $V = 200$. Both $k_1$ and $k_2$ are maximised (i.e., no symmetry breaking). **b.** $V = 500$. The value of $k_1$ is maximised, but that of $k_2$ is minimised (i.e., symmetry breaking). Note that both $k_1$ and $k_2$ first decreases, and $k_1$ starts to increase after $k_2$ decreases sufficiently. This dynamics is similar to that depicted in Fig. 4. **c.** $V = 800$. Both $k_1$ and $k_2$ are minimised (i.e., no symmetry breaking). Parameters: $m = 0.04$, $\delta = 1$, $r = 0.5$, $N = 50V$.

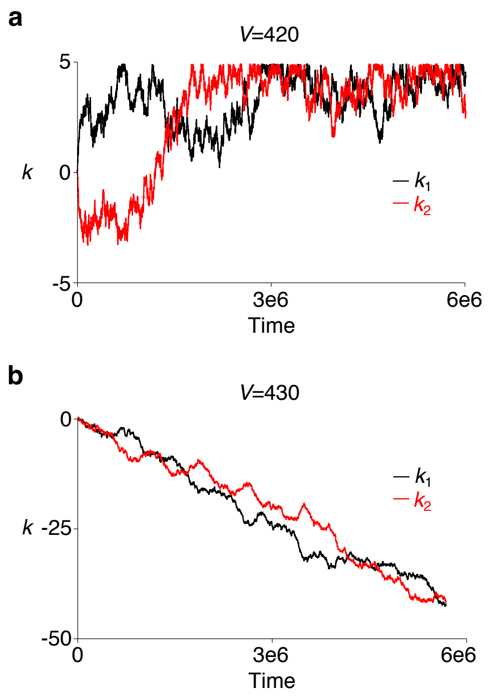


Supplementary Figure 9: The absence of symmetry breaking in the minimal model with the second method of mutation (see Supplementary Methods under 'The second method of mutating $k_x$'). The population averages of $k_x$ values are plotted as functions of time (one unit of time corresponds to $N$ birth-death steps). **a.** $V = 420$. Both $k_1$ and $k_2$ are maximised (i.e., no symmetry breaking). Qualitatively the same result was obtained for $V = 100$, 200, 300, 400, 410 (data not shown). **b.** $V = 430$. Both $k_1$ and $k_2$ are minimised (i.e., no symmetry breaking). Qualitatively the same result was obtained for $V = 430$, 440, 450, $\cdots$, 500, 600, 700, $\cdots$, 1000 (data not shown). Note that the difference between a and b in $V$ is very small. Note also that the model displays symmetry breaking for $V = 500$ if the first method of mutation is employed (Supplementary Fig. 7). Given that the first and second methods of mutation are similar, these results suggest that the model does not display symmetry breaking if the second method of mutation is employed. The parameters were the same as in Supplementary Fig. 7.

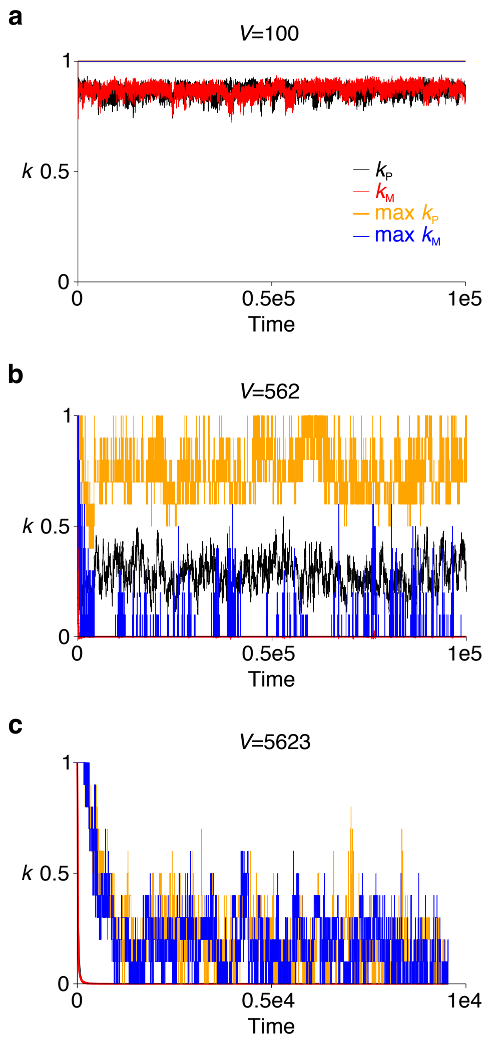


Supplementary Figure 10: Symmetry breaking in the model incorporating the RNA-folding genotype-phenotype map (see Supplementary Methods under 'The model incorporating the RNA-folding genotype-phenotype map'). The averages of $k_{\mathrm{P}}$ (black) and $k_{\mathrm{M}}$ (red) and the maximum value of $k_{\mathrm{P}}$ (orange) and $k_{\mathrm{M}}$ (blue) in the population are plotted as functions of time (one unit of time corresponds to $N$ birth-death steps). **a.** $V = 100$. Both $k_1$ and $k_2$ are maximised (i.e., no symmetry breaking). **b.** $V = 562$. The value of $k_1$ is maximised, but that of $k_2$ is minimised (i.e., symmetry breaking). **c.** $V = 5623$. Both $k_1$ and $k_2$ are minimised (i.e., no symmetry breaking). Parameters: $m = 0.1$, $r = 0.1$, $N = 50V$. The target structure ($p_0$) was set to '`......(((....))))....(((..((((((((....)))))))))))....`' in the dot-bracket notation. The population was initialised with sequence `GAAAGCCUGAAUCCAGAGAGACCCAGUGGCAGGACGUUGUUACGGUAAGA` and its complementary sequence, both of which fold into the target structure. See Supplementary Fig. 11 for how the target structure and initial sequences were selected.

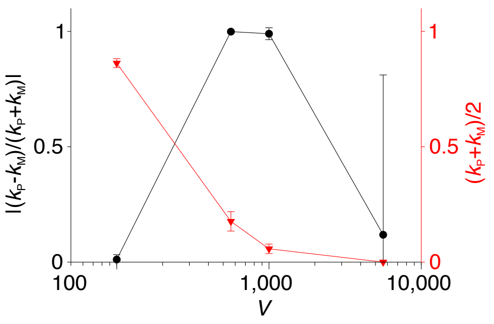


Supplementary Figure 11: Symmetry breaking in the model incorporating the RNA-folding genotype-phenotype map for many target structures (see Supplementary Methods under 'The model incorporating the RNA-folding genotype-phenotype map'). The model was run with 28 distinct target structures (the parameters were the same as in Supplementary Fig. 10). In each simulation, the degree of functional asymmetry $|(k_{\rm P} - k_{\rm M})|/|(k_{\rm P} + k_{\rm M})|$ and average cooperativeness $(k_{\rm P} + k_{\rm M})/2$ of replicators were averaged over time after equilibration. The mean (symbols) and s.d. (error bars) for the 28 target structures are plotted. Colour coding: $|(k_{\rm P} - k_{\rm M})|/|(k_{\rm P} + k_{\rm M})|$ (black circle, left coordinate), $(k_{\rm P} + k_{\rm M})/2$ (red triangle, right coordinate). The results show that the degree of functional asymmetry increases for an intermediate range of $V$, indicating the robustness of the symmetry breaking in this model. The target structures were generated as follows: 32 RNA sequences were randomly generated (each base with an equal probability). The sequences were folded with the RNA folding algorithm. For each of the 32 structures obtained, a genetic algorithm was used to obtain a pair of complementary sequences both of which fold into the structure (Fisher-Wright process; random initial population of size 2000; $m = 1$). Four of the 32 structures did not yield such sequences within an arbitrarily-chosen number of generations and were discarded. The remaining 28 structures were selected as the target structures, together with the sequences obtained through the genetic algorithm as the initial sequences.

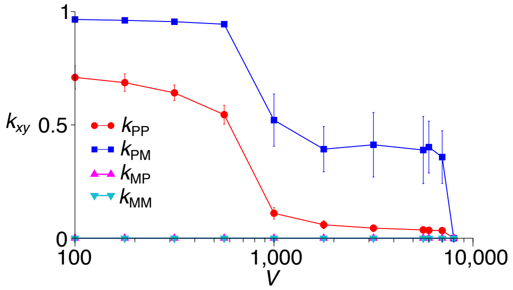


Supplementary Figure 12: Kinetic symmetry breaking in the model on which functional asymmetry is a priori imposed by setting $k_{\mathrm{MP}} = k_{\mathrm{MM}} = 0$ throughout simulations. The equilibrium average catalytic activities ($k_{xy}$) are plotted as functions of cell size ($V$). The values of $k_{xy}$ were first averaged over all replicators at each time point. Then, the average (symbols) and s.d. (error bars) of $k_{xy}$ over time were calculated after equilibration. The parameters were the same as in Fig. 2.

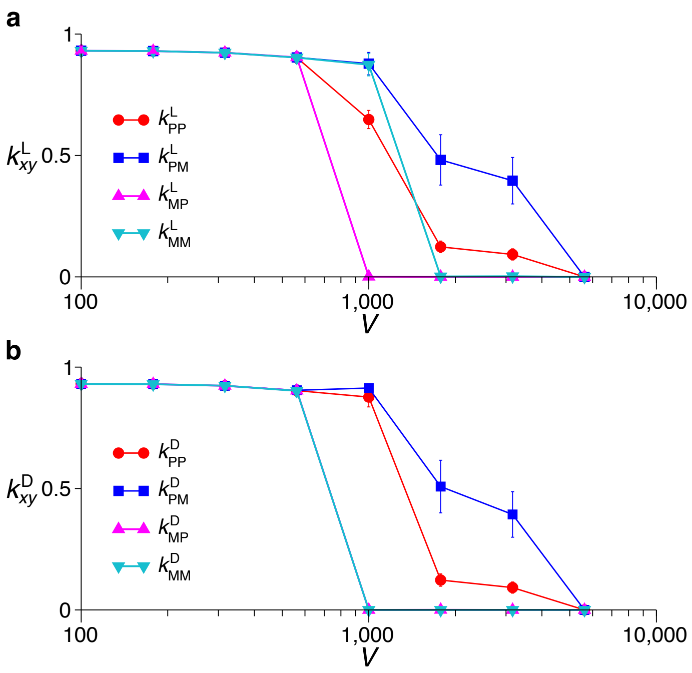


Supplementary Figure 13: Symmetry breaking in the model incorporating the cross-chiral replicators envisaged in the Sczepanski-Joyce experiment (see Discussion for details). The equilibrium average catalytic activities ($k_{xy}$) are plotted as functions of cell size ($V$). The values of $k_{xy}$ were first averaged over replicators for each member of the hypercycle at each time point. Then, the average (symbols) and s.d. (error bars) of $k_{xy}$ over time were calculated after equilibration. **a.** The $k_{xy}$ values of L isomers (i.e., one member of a hypercycle). **b.** The $k_{xy}$ values of D isomers (i.e., the other member of the hypercycle).
